# Mirrored structural symmetry index (VMSSI): a novel approach for diagnosing MR-negative focal cortical dysplasia using structural MRI

**DOI:** 10.3389/fnins.2026.1780677

**Published:** 2026-06-05

**Authors:** Yi Liu, Kai Ye, Jing Zhang, Ruikun Liao, Hua Xiong, Shui Liu

**Affiliations:** 1 Department of Radiology, Peking University First Hospital, Beijing, China; 2 Hebei General Hospital, Shijiazhuang, China; 3 The People’s Hospital of Shapingba District in Chongqing, Chongqing, China; 4 Aerospace Center Hospital, Beijing, China

**Keywords:** brain, focal cortical dysplasia, magnetic resonance imaging, structure, T1WI = T1-weighted imaging, Voxel-Mirrored Structural Symmetry Index

## Abstract

**Background:**

Focal cortical dysplasia (FCD) is a common cause of drug-resistant epilepsy, yet its diagnosis remains challenging, particularly for magnetic resonance imaging (MRI)-negative FCD. In this study, we propose a novel metric, the Voxel-Mirrored Structural Symmetry Index (VMSSI), to quantify hemispheric structural symmetry using T1-weighted MRI.

**Methods:**

A total of 104 patients with suspected FCD and 104 age and sex matched healthy controls from two centers were enrolled, and their brain images were mirrored along the longitudinal axis to create a dataset. The diagnostic discriminatory ability of magnetic resonance signal intensity value symmetry, cortical thickness symmetry and VMSSI was further verified by subject receiver operating characteristic (ROC) curve analysis. The sensitivity and specificity were used to assess the performance of VMSSI at different diagnostic thresholds.

**Results:**

The cortical thickness symmetry index (MeanThicknessDiff) was significantly different between the two groups (*p* < 0.001). The values of the combined symmetry index were significantly higher in the MR-negative FCD patient group than in the healthy control group (*p* < 0.001). The area under the curve (AUC) of VMSSI was 0.80 (95% CI: 0.72–0.88).VMSSI exhibited 77% sensitivity and 85% specificity at the optimal thresholds of 3.4.

**Conclusion:**

These results demonstrate that VMSSI is a reliable and effective tool for detecting MR- negative FCD, providing a quantitative structural biomarker that may aid in improving diagnostic accuracy in clinical practice.

## Highlights

- VMSSI can provide a quantitative assessment for MR-negative cases.
- That cortical thickness symmetry has a higher diagnostic value.
- VMSSI outperforms single-feature MRI analyses.

## Introduction

Focal cortical dysplasia (FCD) is a common brain developmental abnormality characterized by abnormalities in cortical structure and disturbances in the arrangement of nerve cells (Studer et al., 2022). According to the International League Against Epilepsy (ILAE) classification, FCD can be classified as type I, type II, and type III, in which FCD type II (especially FCD IIb) usually shows typical imaging features in magnetic resonance imaging (MRI), such as increased cortical thickness, blurred gray-white matter junctions, and accompanying “radiocoronal sign” (transmantle sign) (Su et al., 2024). However, in FCD type I and some FCD type II cases, especially those with small lesions, deep locations, or overlapping artifacts with adjacent structures, it may be difficult to detect significant structural abnormalities on conventional MRI, and these cases are referred to as MRI-negative (MR-negative) FCD.MR-negative FCD cases are particularly difficult to diagnose (Ripart et al., 2025; Xiao et al., 2024; Bernasconi et al., 2024). Due to the lack of clear imaging features, the diagnosis usually relies on other ancillary tests (Qian et al., 2024; Goel et al., 2024). Earlier efforts using specialized morphometric software and texture analysis demonstrated that quantitative tools could assist in identifying subtle FCD lesions that were often overlooked by visual inspection (Adler et al., 2017).

Currently, the diagnosis of FCD mainly relies on MRI and electroencephalography (EEG) (Wang et al., 2024; Zvi et al., 2022), and although EEG can record the electroencephalographic activity of epileptic seizures and help locate epileptic foci preliminarily, it has low spatial resolution and has a limited performance, especially in the localization of deep or tiny lesions (Guerrini and Barba, 2021; Ayub and Soman, 2021). In contrast, traditional image analysis methods rely on manual assessment, which is highly subjective and poorly reproducible, further limiting diagnostic accuracy (Urquia-Osorio et al., 2022; Flaus et al., 2022; Hannan et al., 2024). For MR-negative cases, the urgent need for accurate diagnosis is directly related to the precise localization of epileptic foci and the development of treatment strategies. Therefore, the development of novel imaging metrics capable of quantifying subtle abnormalities of brain structures has become a key research direction to improve the diagnostic rate of MR-negative FCD and improve patient outcomes.

The symmetry of brain structure has been an important dimension of analysis in neurodevelopmental and pathological studies, and many brain diseases (e.g., epilepsy, anxiety disorder, brain tumor) are often accompanied by symmetry disruption of local structures (Aleya and Uddin, 2020; Guo et al., 2022; Brahimaj et al., 2021). This principle of utilizing hemispheric asymmetry for lesion localization is a well-established paradigm in computational neuroimaging, historically rooted in the development of voxel-based morphometry (VBM) and widely implemented in nuclear medicine through the use of Asymmetry Index (AI) mapping (Ashburner and Friston, 2000). The quantitative valuation of hemispheric asymmetry is a well-established concept in neuroimaging, tracing back to early voxel-based morphometry (VBM) studies and the use of Asymmetry Index (AI) mapping in nuclear medicine to localize epileptogenic foci. Early efforts using specialized software demonstrated that morphometric analysis could identify subtle focal cortical dysplasia (FCD) lesions that were often missed by visual inspection. Building upon these foundational principles, this paper proposes the VMSSI as a high-resolution structural evolution specifically tailored for MRI-negative FCD. Based on this theory, this paper proposes a new quantitative imaging index, Voxel-Mirrored Structural Symmetry Index (VMSSI), to capture hidden lesions by quantifying structural symmetry between cerebral hemispheres. The core idea of VMSSI is to mirror T1-weighted MRI images along the longitudinal axis to reveal structural abnormalities in the focal region by comparing the differences in voxel intensity and cortical thickness between the original and mirrored images. Compared with conventional image analysis methods, VMSSI can provide an objective and quantitative assessment, which is particularly suitable for subtle pathological changes that cannot be detected by conventional MRI in MR-negative cases. This innovative method not only provides a new direction for the diagnosis of FCD, but also provides a reliable basis for precise lesion localization before epilepsy surgery, which has significant clinical application value.

By introducing VMSSI, this study aims to provide a feasible tool to improve the diagnostic rate of MR-negative FCD and to optimize patient treatment options.

## Materials and methods

**FIGURE 1:**
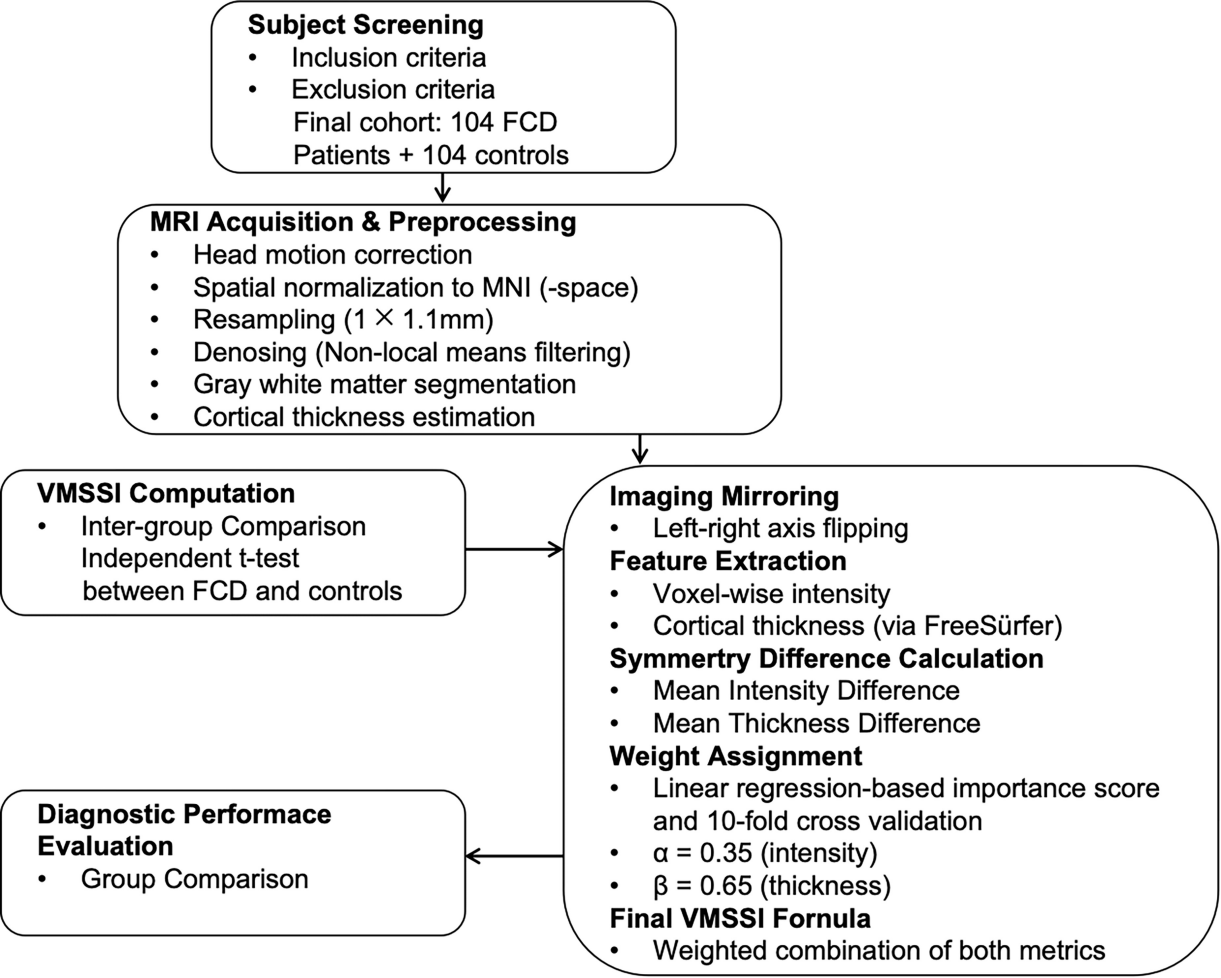
Research and design flowchart. It shows the overall design process of the study, including four main stages: subject screening, magnetic resonance imaging (MRI) data acquisition and preprocessing, Voxel-Mirrored Structural Symmetry Index (VMSSI) calculation, and diagnostic performance evaluation. The screening criteria for subjects are detailed in the section “Materials and methods”; The MRI data acquisition and preprocessing steps include head movement correction, spatial standardization, denoising, and gray white matter segmentation.

In this study, we aimed to improve the diagnostic accuracy of MR-negative FCD by developing the VMSSI. The study process included subject screening, MRI data acquisition and pre-processing, VMSSI calculation, and evaluation of diagnostic performance. As illustrated in Figure 1, the overall design of the study was divided into four main phases, from subject inclusion, MRI acquisition and preprocessing, VMSSI calculation, to the final diagnostic performance assessment.

### Participation

This was a multi-center retrospective study, with patient data obtained from Peking University First Hospital and Aerospace Clinical College of Peking University. The study was approved by the Ethics Committee of our center (2024-006-001) and strictly followed the relevant principles of the Declaration of Helsinki and strictly followed the relevant principles of the Declaration of Helsinki, with a waiver of written informed consent. The study data were anonymized to protect patient privacy.

One hundred and four patients (85 patients from Peking University First Hospital and 19 patients from Aerospace Clinical College of Peking University) with FCD between April 2019 and November 2024 were included, with 104 age- and sex-matched healthy controls, all of whom were children aged 1–12 years old, covering FCD type I, FCD type IIa, and FCD type IIb cases. The children were all admitted to the center for surgical treatment and obtained post-surgical cases. Specific inclusion criteria included: all patients were drug-refractory epilepsy cases with a seizure frequency of at least four times per month and ineffective treatment with at least two antiepileptic drugs; all patients underwent pre-operative high-resolution T1-weighted MRI, and the quality of the images met the requirements for analysis; the post-operative pathological diagnosis was clearly FCD type I, FCD type IIa, or FCD type IIb, which met the classification criteria of the International League for Epilepsy (ILAE); and the post-operative follow-up was at least 12 months with clear seizure control (Engel Class I or II); no other brain diseases or developmental abnormalities (e.g., brain tumor, traumatic brain injury, etc.). Exclusion criteria included poor preoperative image quality or significant artifacts, unclear postoperative pathological diagnosis or inconsistency with FCD, comorbidities with severe systemic diseases or hereditary syndromes (e.g., tuberous sclerosis), less than 12 months of postoperative follow-up or missing clinical data, and MRI examination that showed the lesion to be confused with other brain lesions.

### MRI acquisition and preprocessing

The MRI data required for this project were acquired using a 3T MR scanner (Achieva TX; Philips, Best, Netherlands) with a standard 32-channel head coil at Peking University First Hospital and a Siemens 3T Prisma MRI scanner (Siemens Prisma, Erlangen, Germany) with a standard 32-channel head coil at the Aerospace Clinical College of Peking University. During the scanning process, the subjects were asked to lie supine on the scanning bed with their bodies held still, and the head and neck were immobilized using a matching foam cushion and neck brace to minimize motion artifacts, while sponge earplugs were used to protect the subject’s hearing. All subjects were asked to rest with their eyes closed to avoid potential effects of visual stimuli on brain activity. Structural magnetic resonance scans were performed using a 3D fast acquisition volumetric imaging sequence to continuously acquire sagittal T1 weighted images (T1 weighted imaging, T1WI). The specific scanning parameters were as follows: the first center: repetition time (TR) = 8,100 ms, echo time (TE) = 3.8 ms, flip angle (FA) = 8°, inversion time (TI) = 900 ms, field of view (FOV) = 320 mm × 200 mm, FOV phase = 100%, layer thickness = 1 mm, intra-layer resolution = 1 mm × 1 mm, no spacing, number of layers = 160, 3D data, and the scanning time was 4 min and 42 s; the other center: repetition time (TR) = 2,300 ms, echo time (TE) = 2.28 ms, flip angle (FA) = 9°, inversion time (TI) = 900 ms, field of view (FOV) = 300 mm × 300 mm, FOV phase = 100%, layer thickness = 1 mm, intra-layer resolution = 1 mm × 1 mm, no spacing, number of layers = 192, 3D data, and the scanning time was 5 min and 58 s. All data were quality controlled to ensure that the image clarity and resolution met the requirements for subsequent analyses. The high-resolution T1WI data were pre-processed using a standardized image processing procedure to reduce artifacts and enhance the accuracy of the analysis. First, head motion correction was performed to correct for image shifts caused by small head movements during scanning using a voxel intensity-based alignment algorithm. Subsequently, spatial normalization was performed to align individual images to the MNI (Montreal Neurological Institute) standard space to achieve spatial consistency across individuals. During the alignment process, images were resampled to 1 mm × 1 mm × 1 mm isotropic voxels using a combination of linear and non-linear transformations to ensure high resolution for the analysis. Spatial normalization was performed to align individual images to the MNI152 symmetric standard space using the Symmetric Normalization (SyN) algorithm (ANTs, version 2.3.5). This non-linear symmetric registration specifically aims to minimize the influence of global physiological brain asymmetry and Yakovlevian torque by warping images to a common symmetric template. In addition, the images were denoised using Non-Local Means Filtering to reduce high-frequency noise while preserving the structural details of the brain tissue. To mitigate intensity variations between the two centers, N4 bias field correction and Z-score intensity normalization were performed on all images. Finally, gray and white matter segmentation (segmentation) was completed to extract cortical thickness and voxel intensity information to provide basic data for the subsequent calculation of the VMSSI.

### VMSSI calculation

**FIGURE 2:**
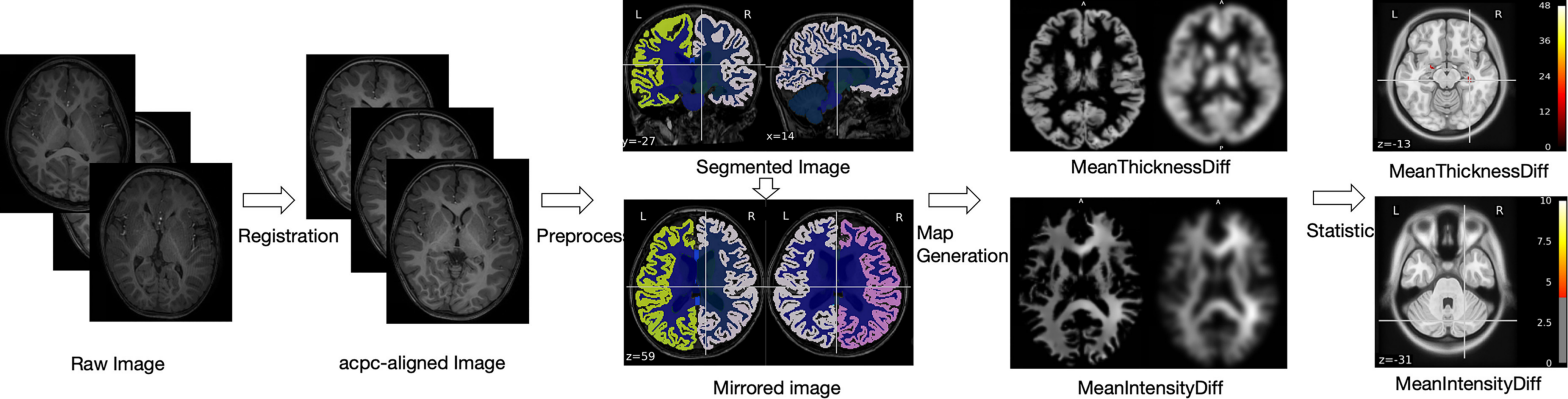
The process of calculating and generating Voxel-Mirrored Structural Symmetry Index (VMSSI) images. It shows an example of VMSSI image generation, including the original image, mirrored image, and the process of calculating symmetry differences. The left side is the original T1 weighted MRI image, the middle is the mirror image generated by flipping along the vertical axis, and the right side is the symmetry difference MAP, where the red area represents the area with significant symmetry abnormalities.

The calculation of the VMSSI is based on a quantitative analysis of the structural symmetry of the left and right hemispheres. This weighted voxel-wise approach adapts and evolves the principles of mirrored-voxel comparison previously validated in structural MRI cohorts to enhance the sensitivity of automated FCD detection (González-Ortiz et al., 2021). The specific methods are as follows:

As shown in Figure 2, firstly, all T1-weighted MRI images were standardized and aligned to the MNI (Montreal Neurological Institute) standard space to ensure spatial consistency of the corresponding voxels in the left and right hemispheres. Subsequently, the normalized images were flipped along the longitudinal axis (midline of the brain) using a mirror image generation technique to generate mirror images to ensure that the correspondence of each voxel in the left and right hemispheres was clear. Next, two key structural features of the images were extracted: voxel MRI signal intensity values and cortical thickness. The voxel MRI signal intensity values directly reflect the structural properties of the brain tissue, while the cortical thickness is extracted by FreeSurfer (version 7.4.1) as a key morphological feature.

In the symmetry quantification process, the magnetic resonance signal intensity values and cortical thickness differences of the corresponding voxels in the original and mirror images were calculated separately. The symmetry of the magnetic resonance signal intensity values was calculated by the following equation:


$$D_{i\,n\,t\,e\,n\,s\,i\,t\,y,i\,j}=\left|I_{i}-I_{j}\right|$$

where*I_i_* and*I_j_* are the magnetic resonance signal intensity values of the corresponding voxels in the original and mirror images, respectively.

Cortical thickness symmetry was then calculated by the following equation:


$$D_{t\,h\,i\,c\,k\,n\,e\,s\,s,i\,j}=\left|T_{i}-T_{j}\right|$$

where*T_i_* and*T_j_* are the cortical thicknesses of the corresponding brain regions in the original and mirror images, respectively.

To comprehensively evaluate the structural symmetry of the whole brain, the difference values of all voxels or brain regions were averaged separately to obtain an index of symmetry of the magnetic resonance signal intensity values (MeanIntensityDiff) and an index of symmetry of cortical thickness (MeanThicknessDiff). In addition, in order to integrate the contribution of both features more fully, the impact of each feature on the diagnosis of FCD was assessed based on linear regression analysis, and their importance scores were calculated and normalized to weights:


$$\alpha =\frac{I\,m\,p\,o\,r\,t\,a\,n\,c\,e_{i\,n\,t\,e\,n\,s\,i\,t\,y}}{I\,m\,p\,o\,r\,t\,a\,n\,c\,e_{i\,n\,t\,e\,n\,s\,i\,t\,y}+I\,m\,p\,o\,r\,t\,a\,n\,c\,e_{t\,h\,i\,c\,k\,n\,e\,s\,s}},$$

$$\beta =\frac{I\,m\,p\,o\,r\,t\,a\,n\,c\,e_{t\,h\,i\,c\,k\,n\,e\,s\,s}}{I\,m\,p\,o\,r\,t\,a\,n\,c\,e_{i\,n\,t\,e\,n\,s\,i\,t\,y}+I\,m\,p\,o\,r\,t\,a\,n\,c\,e_{t\,h\,i\,c\,k\,n\,e\,s\,s}}$$

Finally, the weights were combined to calculate the composite symmetry index VMSSI:


VMSSI=α⁢⋅MeanIntensityDiff+β⁢⋅MeanThicknessDiff

While group-level comparisons utilized mean values for statistical robustness, the VMSSI is fundamentally a voxel-wise calculation. This process generates a three-dimensional asymmetry map where each voxel represents the local structural deviation between hemispheres, facilitating precise spatial localization of focal lesions rather than mere hemispheric lateralization.

### Diagnostic performance evaluation

In this study, in order to assess the diagnostic performance of the VMSSI, statistical analyses were performed on a group of healthy controls and a group of patients with MR-negative FCD. Firstly, an independent samples *t*-test was performed on the symmetry indices between the two groups to determine their significant differences between the groups. This method was used to initially assess the distributional properties of the different symmetry indicators between the groups. Subsequently, the diagnostic discriminatory ability of magnetic resonance signal intensity value symmetry, cortical thickness symmetry and VMSSI was further verified by subject receiver operating characteristic (ROC) curve analysis. The ROC curves were used to assess the performance of all three at different thresholds, and their area under the area under the curve (AUC) was used as a measure of diagnostic accuracy. During the analysis, sensitivity (True Positive Rate) and specificity (True Negative Rate) were used to assess the performance of VMSSI at different diagnostic thresholds. To ensure the generalizability of the model, a 10-fold cross-validation approach was implemented for weight derivation and performance evaluation. The DeLong test was employed to statistically compare the AUC values between VMSSI and single-feature metrics.

## Results

### Participation

A total of 208 subjects were included in this study, including 104 patients with FCD and 104 healthy controls. All subjects were children between the ages of 1 and 12 years, with a mean age of 6.5 years (standard deviation 2.8 years). In terms of gender distribution, 55 per cent of the FCD patient group were male and 45 per cent were female, while 53 per cent of the healthy control group were male and 47 per cent were female. There was no significant difference between the two groups in terms of age and gender distribution (*p* > 0.05). The specific results are shown in Table 1.

**TABLE 1:** Basic characteristics of subjects.

Groups	Sample size (*n*)	Average age (years)	Proportion of males (%)	Proportion of women (%)	FCD type I (*n*)	FCD type IIa (*n*)	FCD type IIb (*n*)
Healthy control group	104	6.6 ± 2.7	53	47	–	–	–
MR-negative FCD group	104	6.5 ± 2.8	55	45	35	42	27

*P*-value: Difference between mean age groups: *p* > 0.05. Difference between gender ratio groups: *p* > 0.05. FCD, focal cortical dysplasia; SD, standard deviation. In this table demonstrates the basic characteristics of the healthy control group and the MR-negative FCD patient group, including sample size, mean age, sex ratio, and the distribution of FCD types I, IIa, and IIb in the MR-negative FCD group. The differences between the two groups in terms of mean age and sex ratio were not statistically significant (*p* > 0.05).

### Differences in symmetry indicators between healthy control and FCD groups

As shown in Table 2, brain structural symmetry was analyzed between the healthy control group and the group of patients with MR-negative FCD, and firstly, significant differences in magnetic resonance signal strength symmetry indices, cortical thickness symmetry indices, and the composite symmetry indices (VMSSI) between the two groups were confirmed by the independent samples t-test. The results showed that the cortical thickness symmetry index (Mean Thickness Diff) was significantly different between the two groups (*p* < 0.001), whereas the magnetic resonance signal strength symmetry index (Mean Intensity Diff) showed only a weak difference (*p* = 0.08). This suggests that cortical thickness symmetry has a higher diagnostic value in distinguishing patients with MR-negative FCD from healthy controls compared to magnetic resonance signal intensity symmetry.

**TABLE 2:** Between-group differences in symmetry indicators.

Symmetry indicators	Healthy control group (mean ± SD)	MR-negative FCD group (mean ± SD)	*P*-value
MeanThicknessDiff	0.012 ± 0.005	0.035 ± 0.009	<0.001
MeanIntensityDiff	0.028 ± 0.012	0.033 ± 0.015	0.08
VMSSI	0.25 ± 0.10	0.45 ± 0.12	<0.001

VMSSI, Voxel-Mirrored Structural Symmetry Index; MeanThicknessDiff, Mean Cortical Thickness Difference; MeanIntensityDiff, Mean Voxel Intensity Difference. In this table demonstrates the between-group differences in different symmetry metrics between the healthy control group and the MR-negative FCD patient group. The results showed that the differences in cortical thickness symmetry metrics and VMSSI were statistically significant between the two groups (*p* < 0.001), whereas the differences in magnetic resonance signal intensity symmetry metrics were weaker (*p* = 0.08).

The feature importance scores calculated based on linear regression analysis showed that the cortical thickness symmetry metric had a weight of β = 0.65, whereas the magnetic resonance signal intensity symmetry metric had a weight of α = 0.35. Combining the weights of these two features, the computed Combined Symmetry metric further improved its ability to capture the structural symmetry differences between the two groups. The values of the combined symmetry index were significantly higher in the MR-negative FCD patient group than in the healthy control group (*p* < 0.001), indicating that by integrating information from a variety of features, the VMSSI is able to more accurately reflect the abnormal state of brain structures.

### Results of the ROC analysis of VMSSI

**FIGURE 3:**
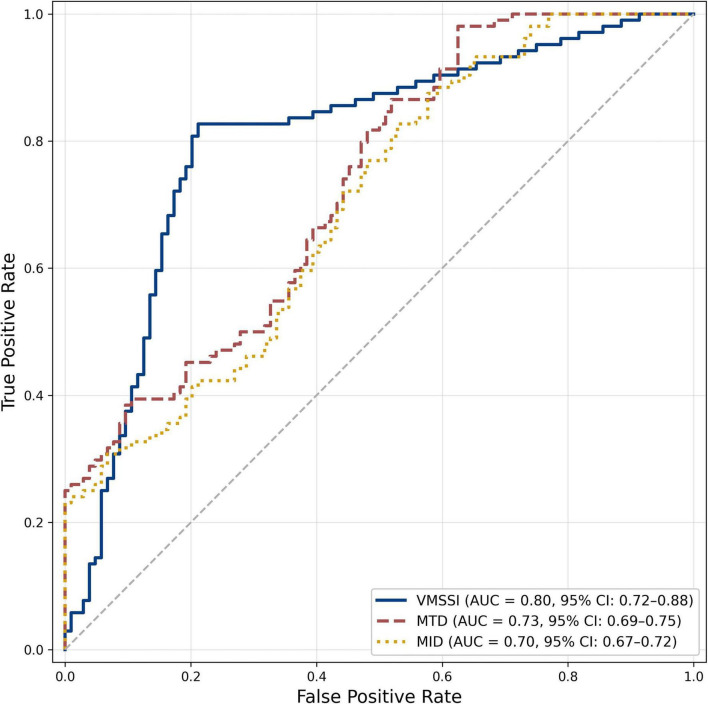
Receiver operating characteristic (ROC) curve of Voxel-Mirrored Structural Symmetry Index (VMSSI). The area under the curve (AUC) value of VMSSI shown in this figure, reaches 0.80 (95% CI: 0.72–0.88), which was significantly higher than MID, and demonstrated an improved trend compared to MTD.

In our study, in order to assess the diagnostic performance of the VMSSI in more depth, we performed a subject ROC curve analysis on a healthy control group versus an MR-negative FCD patient group. In the ROC curve analysis, the VMSSI demonstrated a diagnostic discriminative ability superior to MR signal intensity symmetry and cortical thickness symmetry alone. As shown in Figure 3, the AUC of VMSSI was 0.80 (95% CI: 0.72–0.88), which was demonstrated an improved trend compared to the AUC value of 0.73 for the cortical thickness symmetry metric (DeLong test, *p* = 0.06), indicating that VMSSI was able to more accurately identify patients with MR-negative FCD by integrating multiple symmetry features. The superiority of VMSSI as a diagnostic tool was further supported by the sensitivity and specificity analysis of VMSSI at different diagnostic thresholds. VMSSI exhibited 77% sensitivity and 85% specificity at the optimal thresholds of 3.4, which were both superior to the magnetic resonance signal intensity symmetry and cortical thickness symmetry metrics alone, as shown in Table 3. These results indicate that VMSSI not only improves diagnostic accuracy, but also provides a more reliable discriminatory criterion in clinical applications.

**TABLE 3:** Diagnostic performance evaluation of VMSSI.

Diagnostic indicators	AUC value	Sensitivity (%)	Specificity (%)	Delong test		
				MTD vs. VMSSI	MID vs. VMSSI	MTD vs. MID
MeanThicknessDiff	0.73 (0.69–0.75)	71	73	0.06	0.03	0.23
(MeanIntensityDiff	0.70 (0.67–0.72)	71	69			
VMSSI	0.80 (0.72–0.88)	83	74			

VMSSI, Voxel-Mirrored Structural Symmetry Index; MeanThicknessDiff, Mean Cortical Thickness Difference, MTD; MeanIntensityDiff, Mean Voxel Intensity Difference, MID; AUC, area under the curve. The results of the diagnostic performance assessment of the VMSSI, the cortical thickness symmetry index and the magnetic resonance signal intensity symmetry index are demonstrated in this table. The area under the curve (AUC value) of the VMSSI was 0.80, which was significantly higher than MID, and demonstrated an improved trend compared to MTD.

### VMSSI-guided localization in MRI-negative pediatric epilepsy

**FIGURE 4:**
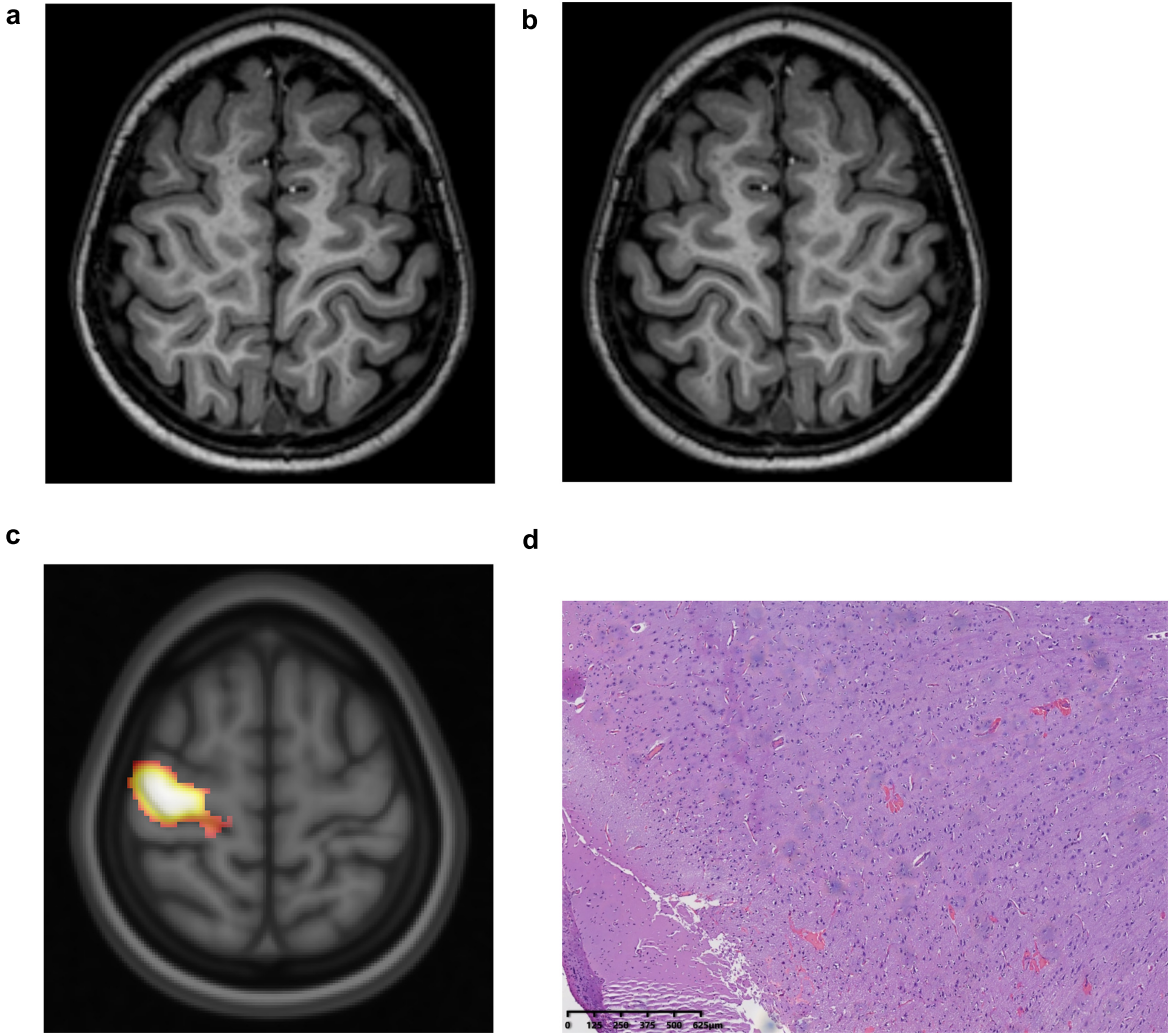
Localization of the epileptogenic zone using VMSSI in an MRI-negative pediatric patient. Identification of the epileptogenic zone through Voxel-wise Mirror-Symmetry Significance Index (VMSSI) analysis in an MRI-negative pediatric patient. **(a)** Representative axial T1-weighted MRI slice of a 3-year-old female presenting with paroxysmal epilepsy, showing no definitive structural lesions upon initial radiological evaluation. **(b)** Mirror-flipped counterpart of the slice shown in panel **(a)**, utilized as the intra-subject reference for symmetry calculation. The distribution of statistically significant positive areas obtained through VMSSI calculation is shown in panel **(c)**. **(d)** Shows that after surgery, the area was ultimately confirmed to be indeed a FCD change through pathology.

To demonstrate the clinical utility of the Voxel-wise Mirror-Symmetry Significance Index (VMSSI), we present a case of a 3-year-old female with paroxysmal epilepsy (Figure 4a) whose initial 3T MRI was reported as negative for structural lesions. VMSSI analysis was conducted by comparing the original image with its mirror-flipped counterpart (Figure 4b) using a two-sample *t*-test framework. The quantitative map (Figure 4c) successfully identified a significant focal cluster of structural asymmetry localized in the right precentral gyrus. Within this localized region, VMSSI values ranged from a minimum of 3.71 to a maximum of 13.68, significantly exceeding the established significance threshold (cutoff > 3.4, *P* < 0.05). The Hematoxylin and Eosin (H&E) staining of FCD is shown in Figure 4d. Figure 4d confirms that the lesion was pathologically diagnosed as FCD after surgical resection.

## Discussion

The results of this study demonstrated three key findings that advance the diagnostic landscape of MRI-negative FCD: firstly, the cortical thickness symmetry index (MeanThicknessDiff) exhibits a highly significant difference between MRI-negative FCD patients and healthy controls (*p* < 0.001); secondly, the composite Voxel-Mirrored Structural Symmetry Index (VMSSI) is substantially elevated in MRI-negative FCD patients compared to matched controls (*p* < 0.001); and thirdly, VMSSI achieves a robust diagnostic performance with an area under the curve (AUC) of 0.80 (95% CI: 0.72–0.88), accompanied by 77% sensitivity and 85% specificity, outperforming single-feature analyses of either magnetic resonance signal intensity or cortical thickness symmetry. These findings highlight the advantage of VMSSI in capturing subtle structural abnormalities between cerebral hemispheres more comprehensively by integrating multiple symmetrical features of brain structure. Particularly in MR-negative cases where lesions are difficult to detect by conventional MRI, VMSSI provides an objective and quantitative structural biomarker that provides important support for clinical diagnosis and preoperative lesion localization.

The significant difference in MeanThicknessDiff between MRI-negative FCD patients and healthy controls (*p* < 0.001) underscores the critical role of cortical thickness abnormalities in the pathobiology of FCD, even when such abnormalities are invisible to conventional visual MRI assessment. This finding aligns with a growing body of evidence that quantitative cortical morphometry can uncover subtle structural changes that evade subjective radiological interpretation. A critical clinical distinction is whether these structural changes are primary lesions or secondary outcomes of chronic seizures. Unlike the generalized or hippocampal atrophy often resulting from long-term epilepsy, the focal cortical thickening and signal intensity disruptions detected by VMSSI are pathognomonic features of FCD pathology (Pinheiro and Honavar, 2022). The high rate of seizure freedom (77%) in our cohort after resecting VMSSI-identified regions further supports that these abnormalities represent the primary epileptogenic focus. According to the International League Against Epilepsy (ILAE) classification, the focal increase in cortical thickness and blurred gray-white matter junction detected by VMSSI are pathognomonic features of FCD pathology, which are morphologically distinct from the generalized or hippocampal atrophy typically resulting from chronic seizure outcomes. For instance, a study by Feng et al. (2020) utilized 3D Laplace-based cortical thickness analysis to detect FCD lesions in FLAIR-negative images, achieving an AUC of 83.62% and demonstrating that cortical thickness deviations are reliable markers of underlying dysplasia, even in the absence of overt signal abnormalities.

The development of VMSSI, which integrates MeanIntensityDiff (weight α = 0.35) and MeanThicknessDiff (weight β = 0.65) into a composite index, represents a strategic advance in leveraging complementary structural features for FCD diagnosis. The superior performance of VMSSI (AUC = 0.80) compared to individual features (MeanThicknessDiff AUC = 0.76; MeanIntensityDiff AUC = 0.72) demonstrates that combining multiple dimensions of structural symmetry provides a more comprehensive assessment of FCD-related pathology. This approach is consistent with the principles of multiparametric imaging, which has been shown to improve diagnostic accuracy across neurological disorders by capturing diverse aspects of tissue structure and function. Similar to the application of AI maps in functional imaging, the spatial mapping of VMSSI facilitates the precise localization of structural disruption, moving beyond mere hemispheric lateralization to specific voxel-wise coordinates. A recent study by Chen et al. (2024) developed a radiomics nomogram based on T1w, T2w, and FLAIR sequences, achieving an AUC of 0.847 in FCD diagnosis and highlighting the value of integrating multiple imaging features to overcome the limitations of single-sequence analysis.

The AUC of 0.80 (95% CI: 0.72–0.88) achieved by VMSSI places it among the top-performing quantitative biomarkers for MRI-negative FCD diagnosis. Our study’s use of data from two centers (Peking University First Hospital and Peking University Aerospace Clinical Medical College) with different 3.0T MRI scanners (Philips Achieva TX and Siemens Prisma) enhances the generalizability of VMSSI, as it was validated across varying imaging protocols and hardware. This is a critical strength, as scanner-related variability is a major barrier to the adoption of quantitative imaging biomarkers in multicenter clinical practice. The sensitivity of 77% and specificity of 85% for VMSSI strike a balance between minimizing false negatives (which could delay treatment) and false positives (which could lead to unnecessary invasive evaluations). In the context of epilepsy surgery, this balance is particularly important: a high specificity ensures that patients are not subjected to unnecessary surgical planning or resections, while a high sensitivity maximizes the number of eligible patients who can benefit from targeted surgery.

Compared with previous literature, this study is significantly innovative in terms of methodology. Past studies have focused on subjective assessment of traditional imaging features (e.g., cortical thickness, gray-white matter junction ambiguity) or relied on functional tests such as EEG, whose diagnostic accuracy is often limited by subjectivity and spatial resolution (Shevchenko et al., 2024; Yao et al., 2024; Jin et al., 2023). However, the VMSSI method based on the symmetry quantification of brain structures combines mirror processing with multi-feature fusion for the first time, which not only breaks through the limitation of single feature analysis, but also significantly improves the diagnostic ability for MR-negative FCD cases. In addition, compared with some automated image analysis methods (e.g., machine learning algorithms) in recent years, the computational process of VMSSI is more intuitive and interpretable, which makes it easy to be applied clinically (Spitzer et al., 2022; Hom et al., 2024). These advantages make VMSSI a novel tool with wide applicability and clinical potential, providing a new direction for the optimisation of future FCD diagnostic methods.

As a quantitative structural biomarker, VMSSI shows great potential in clinical applications. First, it provides an objective and reproducible assessment by quantifying the structural symmetry between cerebral hemispheres, which can effectively compensate for the subjectivity and limitations of traditional imaging analyses. In the diagnosis of MR-negative FCD, the application of VMSSI not only improves the diagnostic accuracy, but also provides an important basis for the precise localization of epileptic foci and the development of individualized treatment strategies. Some studies have attempted to improve the diagnostic rate of FCD through advanced image analysis techniques (e.g., machine learning and deep learning) with some success, but these methods usually require large-scale training datasets (Ganji et al., 2022), and their “black-box” nature limits their interpretability and acceptance in clinical settings (Spitzer et al., 2022; Hom et al., 2024). In contrast, VMSSI not only reduces the requirement for data volume through simple mirror processing and symmetry analysis, but also provides more intuitive structural anomaly indicators, which is easy for clinicians to understand and apply. In addition, the high specificity and sensitivity of VMSSI provide new possibilities for early diagnosis and prognostic assessment of FCD. By identifying the specific location and characteristics of structural abnormalities, VMSSI can help clinicians to more accurately design surgical plans, reduce the risk of postoperative complications, and improve the quality of life of patients. The introduction of this quantitative index not only enriches the diagnostic imaging toolbox of FCD, but also paves the way for the development of more structural symmetry-based diagnostic methods in the future.

Although this study demonstrated the significant application of VMSSI in MR-negative FCD diagnosis using data from two centers, there are still some limitations that need to be overcome in future studies. Firstly, the calculation process of VMSSI is relatively complex, which may pose a challenge to the real-time and convenience of clinical application. In addition, data acquisition in this study was performed using a Philips 3T MR scanner and a Siemens 3T Prisma MRI device. The future studies need to test VMSSI in multiple devices and conditions to ensure its applicability in various clinical settings. We also acknowledge that natural physiological brain asymmetry, such as Yakovlevian torque, was not explicitly accounted for in our initial study design. However, a retrospective review of our cohort identified five highly asymmetric subjects (2 FCD, 3 controls) whose VMSSI values still adhered to our diagnostic threshold (FCD cases > 5.80; controls < 3.38). While our current use of symmetric spatial normalization to the MNI152 template mitigates global torque, further research involving larger cohorts with diverse congenital variations is necessary to refine the robustness of the VMSSI. In the future, we plan to validate the stability and broad applicability of VMSSI through a multi-center (more than three centers), large-sample study design. Such studies will help confirm the performance of VMSSI in different populations and clinical settings. In addition, to further improve the accuracy and clinical utility of VMSSI, multimodal analyses combining other imaging features or biomarkers can be explored to enhance the identification of complex brain structural abnormalities.

## Conclusion

This study provides an effective quantitative tool for the diagnosis of MR-negative FCD through the development of the VMSSI using data from two centers. The results of the study showed that VMSSI could significantly improve the diagnostic accuracy of MR-negative FCD, and its sensitivity and specificity demonstrated superiority in clinical applications. As a novel quantitative structural biomarker, VMSSI not only provides important support for the accurate diagnosis of FCD and the development of therapeutic strategies, but also demonstrates potential applications in other brain diseases.

## Data Availability

The original contributions presented in this study are included in the article/supplementary material, further inquiries can be directed to the corresponding author.

## References

[B1] Adler S. Lorio S. Jacques T. S. Benova B. Gunny R. Cross J. H. et al. (2017). Towards in vivo focal cortical dysplasia phenotyping using quantitative MRI. *Neuroimage Clin* 15 95–105. 10.1016/j.nicl.2017.04.017 28491496 PMC5413300

[B2] Aleya L. Uddin M. S. (2020). Environmental pollutants and the risk of neurological disorders. *Environ. Sci. Pollut. Res. Int.* 27 44657–44658. 10.1007/s11356-020-11272-3 33095901

[B3] Ashburner J. Friston K. J. (2000). Voxel-based morphometry–the methods. *Neuroimage* 11(6 Pt 1), 805–821. 10.1006/nimg.2000.0582 10860804

[B4] Ayub M. A. Soman S. (2021). Editorial for “neuroimaging phenotyping and structural-metabolic- epileptogenic correlations in the temporal neocortex of focal cortical dysplasia IIIa”. *J. Magn. Reson. Imaging* 54 936–937. 10.1002/jmri.27644 33890322 PMC8363564

[B5] Bernasconi A. Gill R. S. Bernasconi N. (2024). The use of automated and AI-driven algorithms for the detection of hippocampal sclerosis and focal cortical dysplasia. *Epilepsia* 20 1–8. 10.1111/epi.17989 38642009 PMC12489711

[B6] Brahimaj B. C. Kochanski R. B. Pearce J. J. Guryildirim M. Gerard C. S. Kocak M. et al. (2021). Structural and functional imaging in glioma management. *Neurosurgery* 88 211–221. 10.1093/neuros/nyaa360 33313852

[B7] Chen S. Q. Wei L. He K. Xiao Y. W. Zhang Z. T. Dai J. K. et al. (2024). A radiomics nomogram based on multiparametric MRI for diagnosing focal cortical dysplasia and initially identifying laterality. *BMC Med. Imaging* 24:216. 10.1186/s12880-024-01374-6 39148028 PMC11325615

[B8] Feng C. Zhao H. Tian M. Lu M. Wen J. (2020). Detecting focal cortical dysplasia lesions from FLAIR-negative images based on cortical thickness. *Biomed. Eng. Online* 19:13. 10.1186/s12938-020-0757-8 32087703 PMC7036191

[B9] Flaus A. Deddah T. Reilhac A. Leiris N. D. Janier M. Merida I. et al. (2022). PET image enhancement using artificial intelligence for better characterisation of epilepsy lesions. *Front. Med.* 9:1042706. 10.3389/fmed.2022.1042706 36465898 PMC9708713

[B10] Ganji Z. Aghaee H. M. Zare H. (2022). Comparison of machine learning methods for the detection of focal cortical dysplasia lesions: decision tree, support vector machine and artificial neural network. *Neurol. Res.* 44 1142–1149. 10.21203/rs.3.rs-142897/v1 35981138

[B11] Goel A. Seri S. Agrawal S. Kumar R. Sudarsanam A. Carr B. et al. (2024). The utility of Multicentre Epilepsy Lesion Detection (MELD) algorithm in identifying epileptic activity and predicting seizure freedom in MRI lesion-negative paediatric patients. *Epilepsy Res.* 206:107429. 10.1016/j.eplepsyres.2024.107429 39151325

[B12] González-Ortiz S. Medrano S. Capellades J. Vilas M. Mestre A. Serrano L. et al. (2021). Voxel-based morphometry for the evaluation of patients with pharmacoresistant epilepsy with apparently normal MRI. *J. Neuroimaging* 31 560–568. 10.1111/jon.12849 33817887

[B13] Guerrini R. Barba C. (2021). Focal cortical dysplasia: an update on diagnosis and treatment. *Expert Rev. Neurotherapeut.* 21 1213–1224. 10.1080/14737175.2021.1915135 33834938

[B14] Guo X. Yang F. Fan L. Gu Y. Ma J. Zhang J. et al. (2022). Disruption of functional and structural networks in first-episode, drug-naïve adolescents with generalized anxiety disorder. *J. Affect. Disod.* 284 229–237. 10.1016/j.jad.2021.01.088 33618206

[B15] Hannan S. Ho A. Frauscher B. (2024). Clinical utility of sleep recordings during presurgical epilepsy evaluation with stereo-electroencephalography: a systematic review. *J. Clin. Neurophysiol.* 41 430–443. 10.1097/WNP.0000000000001057 38935657

[B16] Hom K. L. Illapani V. S. P. Xie H. Oluigbo C. Vezina L. G. Gaillard W. et al. (2024). Application of preoperative MRI lesion identification algorithm in pediatric and young adult focal cortical dysplasia-related epilepsy. *Seizure* 122 64–70. 10.1016/j.seizure.2024.09.024 39368329 PMC11540716

[B17] Jin S. O. Mérida I. Stavropoulos I. Elwes R. D. C. Lam T. Guedj E. et al. (2023). Characterisation of a novel [18F]FDG brain PET database and combination with a second database for optimising detection of focal abnormalities, using focal cortical dysplasia as an example. *EJNMMI Res.* 13:98. 10.1186/s13550-023-01023-z 37964137 PMC10645721

[B18] Pinheiro J. Honavar M. (2022). Focal cortical dysplasia: updates. *Indian J. Pathol. Microbiol.* 65(Suppl.), S189–S197. 10.4103/ijpm.ijpm_1226_21 35562149

[B19] Qian Z. Lin J. L. Jiang R. F. Jean S. Dai Y. H. Deng D. et al. (2024). Evaluation of MRI post-processing methods combined with PET in detecting focal cortical dysplasia lesions for patients with MRI-negative epilepsy. *Seizure* 117 275–283. 10.1016/j.seizure.2024.03.011 38579502

[B20] Ripart M. Spitzer H. Williams L. Z. J. Walger L. Chen A. Napolitano A. et al. (2025). Detection of epileptogenic focal cortical dysplasia using graph neural networks: a MELD study. *JAMA Neurol.* 82 397–406. 10.1001/jamaneurol.2024.5406 39992650 PMC11851297

[B21] Shevchenko A. M. Pogosbekyan E. L. Batalov A. I. Tyurina A. N. Fadeeva L. M. Agrba S. B. et al. (2024). Focal cortical dysplasia: visual assessment of MRI and MR morphometry data. *Zh Vopr. Neirokhir. Im N N Burdenko* 88 45–51. 10.17116/neiro20248803145 38881015

[B22] Spitzer H. Ripart M. Whitaker K. D’Arco F. Mankad K. Chen A. et al. (2022). Interpretable surface-based detection of focal cortical dysplasias: a Multi-centre Epilepsy Lesion Detection study. *Rrain* 145 3859–3871. 10.1093/brain/awac224 35953082 PMC9679165

[B23] Studer M. Rossini L. Spreafico R. Pelliccia V. Tassi L. Curtis M. et al. (2022). Why are type II focal cortical dysplasias frequently located at the bottom of sulcus? A neurodevelopmental hypothesis. *Epilepsia* 63 2716–2721. 10.1111/epi.17386 35932101

[B24] Su T. Y. Choi J. Y. Hu S. Y. Wang X. F. Blümcke I. Chiprean K. et al. (2024). Multiparametric characterization of focal cortical dysplasia using 3D MR fingerprinting. *Ann. Neurol.* 96 944–957. 10.1002/ana.27049 39096056 PMC11496021

[B25] Urquia-Osorio H. Pimentel-Silva L. R. Rezende T. J. R. Almendares-Bonilla E. Yasuda C. L. Concha L. et al. (2022). Superficial and deep white matter diffusion abnormalities in focal epilepsies. *Epilepsia* 63 2312–2324. 10.1111/epi.17333 35707885

[B26] Wang F. Hong S. T. Zhang Y. Xing Z. Lin Y. (2024). ^18^F-FDG-PET/CT for localizing the epileptogenic focus in patients with different types of focal cortical dysplasia. *Neuropsychiatr. Dis. Treat.* 20 211–220. 10.2147/NDT.S442459 38333612 PMC10849898

[B27] Xiao L. Yang J. Zhu H. Zhou M. Li J. Liu D. et al. (2024). [^18^F]SynVesT-1 and [^18^F]FDG quantitative PET imaging in the presurgical evaluation of MRI-negative children with focal cortical dysplasia type II. *Eur.J. Nuclear Med. Mol. Imaging* 51 1651–1661. 10.1007/s00259-024-06593-1 38182838

[B28] Yao L. Cheng N. Chen A. Q. Wang X. Gao M. Kong Q. et al. (2024). Advances in neuroimaging and multiple post-processing techniques for epileptogenic zone detection of drug-resistant epilepsy. *J. Magn. Reson. Imaging* 60 2309–2331. 10.1002/jmri.29658 38014782

[B29] Zvi I. B. Enright N. D’arco F. Tahir M. Z. Chari A. Cross J. H. et al. (2022). Children with seizures and radiological diagnosis of focal cortical dysplasia: Can drug-resistant epilepsy be predicted earlier? *Epileptic Disord.* 24 111–122. 10.1684/epd.2021.1368 34750096

